# Medical Association of Central New York

**Published:** 1898-12

**Authors:** C. A. Van Der Beek


### Society Proceedings.

### MEDICAL ASSOCIATION OF CENTRAL NEW YORK.

Reported by C. A. VAN DER BEEK, M. D , Secretary.

THE thirty-first annual meeting of the Medical Association of Central New York was held at Music Hall, Auburn, N. Y., October 18, 1898.

The meeting was called to order at 11.05 A. M., by the president, Dr. William C. Krauss, of Buffalo.

Prayer was offered by Rev. George Feld, of Auburn.

Dr. J. P. Creveling, of the committee of arrangements, said :

*Mr. President and Members :*

The Cayuga County Medical Society extends to you today a hearty welcome. Thirty-one years ago the embryo that has developed into this magnificent association was conceived in that society. Today she bids you a welcome return, not as the prodigal, lean and lank, but fully developed and in the full vigor of your manhood. While you remain in this city, gentlemen, you will be the guests of the parent society.

The President—I am sure we all appreciate the kind words of welcome of Dr. Creveling on behalf of the Cayuga County Medical

Society and the committee of arrangements, and we trust that we shall make good use of all that the people of Auburn have offered us today.

The first in order is the reading of the minutes of the previous meeting, but these minutes having been so fully reported in our transactions, the chair will entertain a motion to omit their reading at this time.

Dr. JOHN A. GERIN, of Auburn—I move to omit the reading of the minutes of the last meeting.

Seconded by Dr. Creveling, of Auburn. Carried.

The chair announced the following committees :

*Business Committee*—Drs. B. C. Loveland, Clifton Springs ; Sargent F. Snow, Syracuse ; A. W. Henckell, Rochester.

*Committee on Credentials*—Drs. F. H. Stephenson, Syracuse ; John A. Lichty, Clifton Springs ; F. W. Maloney, Rochester.

*Committee on Ethics*—Drs. H. T. Williams, Rochester ; A. A. Young, Newark ; F. W. Higgins, Cortland.

*Committee on Reception*—John A. Gerin, William S. Cheesman, J. P. Creveling, Frederick Sefton, E. S. Foreman, John O. Palmer, Auburn.

*Committee on Publication*—Drs. William C. Krauss, Buffalo ; C. A. Van der Beek, Rochester ; S. L. Elsner, Rochester.

Dr. E. L. MOONEY, first vice-president, was called to the chair while Dr. William C. Krauss, of Buffalo, delivered the president's address, entitled Degeneracy. Prefacing his address the president made the following remarks :

The thirty-first anniversary of the Medical Association of Central New York finds it in a healthy, robust condition, as evidenced by the unflagging interest shown by your attendance and by the program offered for today's consideration. Thus far its growth has been steadily onward, its membership constantly increasing, its treasury well stocked and its *esprit de corps* unexcelled. Its annual volume of Transactions has been highly commented upon and the scientific work accomplished has been applauded and appreciated. But while we have been so prosperous in the past let us not forget that we owe something to the future and that the high standard of the society can only be maintained by united effort and individual zeal.

It was a prudent move when in 1889 Erie County was admitted to this association. It was, however, but an entering wedge for

Western New York, for today I bring with me the applications of the medical societies of the Counties of Cattaraugus, Chautauqua, Allegany, Niagara and Chemung, desiring affiliation with this association. I would recommend that their delegates be accepted and that these societies become affiliated societies.

With the acceptance of these societies the Counties of Western New York are solidly represented in this association and it seems to me appropriate and judicious, as well as timely, that we appreciate the wisdom of their action, and I would therefore suggest that an amendment be made to Article I. of our Constitution, changing the name of this association from the Medical Association of Central New York to the Medical Association of Central and Western New York.

With the publication of our proceedings today we will have a volume of 500 to 600 pages when bound together. It is the intention of the publication committee to prepare a careful index covering these five years, and a revised Constitution and list of members should be authorised printed to accompany Volume V. I recommend that suitable action be taken with reference to this matter.

Our visit to this beautiful city, to which we have looked forward with much pleasure and anticipation, is unhappily marred by the death of our worthy second vice-president, Dr. E. S. Foreman, who died very suddenly in this city on September 13, 1898. Since 1875 he has been an active member in our midst and a valued member of the present committee on arrangements. Some suitable action on his death might be taken by the society. In conclusion, I wish to thank you heartily for the honor of presiding at this meeting and trust that the work accomplished today will be a fitting preface to that of the coming decade.

The President then proceeded with the scientific part of his address, printed in the JOURNAL for November, 1898.

Dr. GERIN—I move a vote of thanks to Dr. Krauss for his very able, interesting and valuable address on a most important subject, and for the recommendations contained in it, and that the address be referred to a committee of three, appointed by the chair.

Seconded by Dr. Crego, of Buffalo. Carried.

The President then resumed the chair.

The chair appointed a committee on the recommendations of the President to consist of Dr. Weigel, Dr. Jacobson and Dr. Crego, three ex-Presidents of the society.

The following members and delegates registered :

*Cayuga.*

| | | |
|---|---|---|
| Austin, S. E. | Jenkins, J. M. | O'Brien, F. E. |
| Brown, A. H. | Jenkins, N. E. | Palmer, John |
| Cheesman, Wm. S. | Kenyon, Frank | Parker, F. H. |
| Conway, M. P. | Laird, W. R. | Putnam, F. |
| Creveling, J. P. | Lester, E. | Ryan, F. B. |
| Gerin, John | Lewis, LeRoy | Sefton, Frederick |
| Greene, G. | | |

*Cortland.*

Higgins, F. W. Smith, M. R.

*Erie.*

| | | |
|---|---|---|
| Colton, A. J. | Dowd, J. Henry | McAdam, L. J. |
| Congdon, C. E. | Fronczak, F. E. | McGuire, F. W. |
| Crego, F. S. | Krauss, Wm. C. | Wende, G. W. |

*Monroe.*

| | | |
|---|---|---|
| Becker, W. D. | Hordenbrook, E. R. | Roe, John O. |
| Boddy, E. C. | Jameson, Thomas | Sawers, F. H. |
| Boswell, C. O. | Jones, O. E. | Schoonmaker, H. |
| Comfort, C. V. C. | Jones, W. B. | Van der Beek, C. E. |
| Conklin, W. L. | Laur, G. A. | Wallace, W. T. |
| Davis, J. C. | Maier, A. P. | Weaver, John E. |
| Elsner, S. L. | Maloney, F. W. | Weigel, L. A. |
| Graham, H. C. W. | Mason, D. G. | Wolff, W. D. |
| Henckell, A. W. | Mulligan, Wesley | |

*Ontario.*

| | | |
|---|---|---|
| Bishop, E. R. | Lichty, Cora L. | Pratt, John H. |
| Eiseline, D. A. | Lichty, John | Spaulding, F. W. |
| Hallenbeck, O. J. | Loveland, B. C. | Stebbins, F. L. |
| Jewett, J. H. | Merritt, C. P. W. | Thayer, C. C. |

*Seneca.*

Russell, Wm. L. Steinach, Wm.

*Onondaga.*

| | | |
|---|---|---|
| Brown, G. L. | Elsner, H. L. | Kellogg, W. C. |
| Clark, G. E. | Hall, A. L. | Loomis, B. W. |
| Crayton, S. B. | Halsted, T. H. | Marlow, F. W. |
| Cour, A. | Hawley H. B. | Miller, A. B. |
| Easton, F. E. | Jacobson, Nathan | Money, E. L. |

| | | |
|---|---|---|
| Randall, A. B. | Stephenson, Frank H. | Van de Warker, Ely |
| Sears, F. W. | Totman, D. M. | Williams, H. |
| Snow, Sargent F. | Vadeboncoeur, A. F. | Wright, H. B. |

*Oswego.*

| | |
|---|---|
| Dowd, P. M. | Rainier, E. |

*Wayne.*

| | | |
|---|---|---|
| Arnold, J. N. | Hallett, T. H. | Myers, Frank |
| Carmer, M. E. | Lapp, E. H. | Young, A. A. |
| Carr, R. S. | | |

Upon motion the following visitors were invited to be guests of the society and to take part in the discussions:

| | |
|---|---|
| Babbitt, ————, Auburn, | Johnson, W. D., Bergen, |
| Babcock, H. D., Syracuse, | Mengis, Charles, Buffalo, |
| Bellows, George A., Waterloo, | Reynolds, C. J., Buffalo, |
| Bennett, M. L., Watkins, | Robinson, R. W., Auburn, |
| Clark, G. W., Waterloo, | Smith, M. R., McGrawville, |
| Everts, George S., Auburn, | Suiter, A. Walter, Herkimer, |
| Fassett, O. R., Auburn, | White, D. A., Montezuma, |
| Feld, Rev. Geo., Auburn, | Whitney, R. A., Liverpool, |
| Hills, J. E., Willard, | Woodruff, E. G., Auburn. |
| Horton, D. B., Red Creek, | |

Dr. B. C. Loveland, of Clifton Springs, on behalf of the business committee: It has been stated that the time of reading the papers would be limited to ten minutes and the discussion to five minutes, hence in order to get the large number of papers before the meeting, we shall have to adhere very strictly to this rule. The order as set down upon the program will be followed as closely as can be, with the exception that the first presentation will be a case by Dr. Crego, of Buffalo.

Presentation of a case by Dr. Floyd S. Crego, Buffalo.

Milk in the sick-room dietary, by B. C. Loveland, M. D., Clifton Springs.

Tubercular peritonitis, with report of cases cured by laparatomy, by Thomas Jameson, M. D., Rochester.

Discussion of last paper by Dr. Ely Van De Warker, Syracuse; Dr. Nathan Jacobson, Syracuse; Dr. Joseph P. Creveling, Auburn; Dr. J. Henry Dowd, Buffalo; Dr. Charles E. Congdon, Buffalo.

Some practical points in the use of the *x*-ray in medicine and surgery, by L. A. Weigel, M. D., Rochester.

Discussion of the last paper postponed until afternoon session.

The society then adjourned for luncheon, at the invitation of the Cayuga County Medical Society. The afternoon session was convened at 2.30 p. m. The election of officers for the ensuring year being in order, the chair appointed as tellers, Drs. Hallenbeck, of Ontario and Sears, of Onondaga. The officers elected are—president, Dr. E. L. Mooney, of Onondaga; first vice-president, Dr. John Gerin, of Cayuga; second vice-president, Dr. A. L. Beahan, of Ontario; secretary, Dr. C. A. Van der Beek, of Monroe; treasurer, Dr. S. L. Elsner, of Monroe.

The President—I believe we elect a delegate to the American Medical Association. If anyone wishes to go as delegate from this association, I think the secretary can furnish him with the proper credentials.

I will now call for the reports of officers; first that of the secretary.

Dr. Van der Beek—The secretary has no report of importance to make. I can simply give you a little information as to the work of the secretary. There were, at the beginning of this meeting, 240 members, to whom cards were sent calling for papers. There are in the district covered by the sixteen counties represented in the association—in the twenty-one counties now that we expect will be represented—about 1,450 physicians, to whom programs are sent. There were in attendance last year 109 physicians who registered. In addition to sending cards to the 240 members, programs to the 1,450, and transactions to 109 members, about seventy-five letters are written per year, making considerable work which the secretary is very glad to do. The treasurer will give a report regarding the financial condition.

The President—While we are waiting for the treasurer, we might hear the report of the committee on president's recommendations.

The committee on president's address reported as follows: That the recommendations with reference to the admission of the county societies of Cattaraugus, Chautauqua, Allegany, Niagara and Chemung be adopted. Inasmuch as the admission of these counties does not enlarge the territory covered by this association, your committee feels that it would be proper to make this recommendation. In regard to the second recommendation, the committee likewise feels that by the admission of the above-named counties, the territory of the society would warrant a change of name from The Medical Association of Central New York to The Medical Association of Central and Western New York.

The President—These amendments to the Constitution will be voted upon at our next meeting.

Dr. N. Jacobson, Syracuse—Do I understand it requires any amendment for the admission of these counties ? As I read the Constitution it simply states that any societies sending delegates can become members of the association. If New York should send delegates, we would be compelled to admit New York. We have no choice in the matter. As I understand the Constitution, any recognised county society of the state sending delegates to this association has a right to demand admission.

Dr. Ely Van de Warker, Syracuse—The change of name, it strikes me, is a very important matter ; and the name Central and Western New York seems to be a clumsy sort of name, long to write and to refer to. It strikes me as quite a serious matter. A good deal depends upon a name sometimes. Why would it not be a little prudent to go carefully in the matter and refer the matter of the change of name to a committee, where the matter could be given a little more reflection and care ? The subject of euphony, the convenience of reference and the name of a society that has assumed the proportions this has are worthy of consideration.

The President—I desire to state that the matter was given consideration by this committee and this is a committee which has reported on that suggestion, also that the change in name will not be voted upon until the next meeting.

Dr. J. Gerin, Auburn—I believe it is desirable to act upon the suggestion of the committee and notice should be given of a change or proposed amendment to the constitution at this meeting, and then it might be acted upon at the next meeting. I give notice that at the next meeting of this association. I shall offer an amendment to the Constitution changing the name from the Medical Association of Central New York to the Medical Association of Central and Western New York.

Dr. J. N. Arnold, Clyde—I would move that the committee which takes into consideration the amendment to the constitution also take into consideration the subject of change of date. This society meets on the same date as the State Medical Association. There are many members of both associations who cannot attend both. Therefore I would suggest that it take that into consideration.

THE PRESIDENT —If Dr. Arnold will give notice in writing of such a change, it will be acted upon at the Syracuse meeting.

Dr. L. A. WEIGEL, Rochester—It seems to be the general impression that the committee is still in existence. The committee appointed on the president's address has made its report and I presume its labors are ended. If any amendments are to be acted upon, it will be necessary to appoint a new committee for that purpose. There is no committee now. I move that a committee of three be appointed to consider all amendments to the constitution and by-laws and report at the next meeting at Syracuse.

Seconded by Dr. Arnold. Carried.

The chair appointed as such committee Drs. Weigel, Jacobson and Arnold.

Reading of the treasurer's report, by Dr. S. L. ELSNER, Rochester.

Cash in bank, October 19, 1897, $163.87 ; receipts, $138.00 ; total, $301.87.

Disbursements, $186.85 ; cash in bank, October, 17, 1898, $115.02 ; total, $301.87.

The funds of the society have steadily decreased and the expenses increased since I became treasurer. By another year we will have no means of meeting the bills as they come in. Inasmuch as this committee on amendments has been appointed, perhaps it will not be necessary now to say that I would move that an amendment to the constitution be made whereby every member of this association be taxed $1 a year, whether he be present at the meeting or not, and that a bill be rendered him at the end of the year for his dues. That would amount to $250.00 a year. This will enable the society to get along very nicely, even at the rapid rate of increase in its expenses. Inasmuch as the society is growing rapidly and we are constantly enjoying the hospitality of the counties in which we meet, the time will come when it will be really asking too much for those counties to put up such a luxurious lunch as we have had today, and it really ought to come out of the funds of the society. If every member on our books would pay a single dollar as his just dues to the society, whether he be present at the meeting or not, the lunches could very easily be paid out of the general treasury of the society. I will not move that amendment because a committee exists and it will be presented to them in due time and acted upon at the next meeting.

Dr. L. A. Weigel, Rochester—I would like to ask for an interpretation of Article XI. of the Constitution, which says that the annual dues for permanent members and delegates in attendance shall be $1.

The President—The point made by our treasurer is a very good one. Not only those who attend the meetings, but those who belong to the association, should pay their annual dues; and from the reading of Article XI., it seems to me that an amendment to that article might well be made, or that the punctuation of the article be so made as to cover the point in question.

Dr. L. A. Weigel, Rochester—I think the constitution covers the ground now, that every permanent member is liable for his annual dues.

Dr. J. Gerin, Auburn—In order to get at this quickly and save time, I will give notice that I will offer, at the next meeting, an amendment to that part of the constitution, to strike out the words "in attendance," in Article XI.

Dr. L. A. Weigel, Rochester—I move that we approve the report of the treasurer.

Seconded by Dr. S. F. Snow, Syracuse. Carried.

The postponed discussion of Dr. Weigel's paper was then taken up and was discussed by Dr. D. M. Totman, Syracuse; Dr. Gerin, Auburn; and Dr. Hall, Fair Haven. Psoas abscess in women, by Ely Van De Warker, M. D., Syracuse; discussed by Dr. Jacobson, Syracuse. Compound dislocations, by Francis E. Fronczak, M. D., Buffalo. The principles of the radical cure of inguinal hernia, by Wm. B. Jones, M. D., Rochester; discussed by Dr. Creveling, Auburn. Hemianopsia, by F. Higgins, M. D., Cortland. Catarrhal deafness—a more favorable prognosis, by S. F. Snow, M. D., Syracuse; discussed by Dr. John O. Roe, Rochester. Scarlatina, by A. A. Young, M. D., Newark. Creosote or guaiacol as a urinary antiseptic, by J. Henry Dowd, M. D., Buffalo. A clinical study of the relation of the blood, urine and stomach contents in diseases of the stomach, by John A. Lichty, M. D., Clifton Springs. Remarks on extrauterine pregnancy, with report of case, by L. J. McAdam, M. D., of Buffalo.

Dr. A. L. Hall, Fair Haven—I request the privilege at this time to present a memorial of the late Dr. Foreman and to have the same incorporated in the minutes of the transactions of this meeting. I was appointed as a chairman of a committee by our county society to perform that duty, but by reason of other occupation within the last two weeks, I have been unable to prepare it. I wish further time and ask that privilege.

THE PRESIDENT—I think the society will grant the time to Dr. Hall for the preparation of that memorial and have it sent to the Committee on Publication.

Dr. VAN DER BEEK, Rochester—I would announce that the following-named delegates have complied with the constitution of the society and are eligible to election : Drs. G. W. Wende, Erie; A. B. Maine, Wesley Mulligan, F. H. Sawers, J. C. Davis, Geo. A. Lane, O. E. Jones, Monroe ; Drs. A. B. Randall, F. W. Marlow, H. B. Hawley, W. C. Kellogg, Onondaga ; Drs. Cora L. Lichty, D. A. Eiseline, Ontario.

Dr. J. GERIN, Auburn—I would move that they be elected to permanent membership.

Seconded by Dr. Creveling, Auburn. Carried.

Dr. EDWIN R. BISHOP, of Geneva, read a paper in relation to a recent epidemic of small-pox.

Dr. B. C. LOVELAND, Clifton Springs, read the following papers by title : The methods and results of state control in medicine, by William Warren Potter, M. D., Buffalo. Syphilis of the nervous system, by Floyd S. Crego, M. D., Buffalo. Can syphilis be cured ? by A. E. Diehl, M. D., Buffalo. A report of fifty-six consecutive vaginal sections for diseases of the uterus and appendages, with some remarks upon the method described by Pryor for examination of the pelvic contents, and exhibition of instruments, by H. T. Williams, M. D., Rochester. Some cases of plastic surgery, by Wm. S. Cheesman, M. D., Auburn. Résumé of 350 post mortem examinations, by A. W. Henckell, M. D., Rochester, coroner's physician of Monroe county. Report of two cases of scolecitis simulating biliary colic, by A. L. Benedict, M. D., Buffalo. Report of a case, by Frederick Sefton, M. D., Auburn. The necessity of early diagnosis in malignant pelvic disease, by C. C. Frederick, M. D., Buffalo.

Dr. B. C. LOVELAND, Clifton Springs—I move that these papers be requested for publication in the transactions.

Seconded by Dr. Young, Newark. Carried.

Dr. S. L. ELSNER, Rochester—At this time as the meeting is drawing to a close and the hour is getting late, I would move that the society thank our brethren of Auburn for the very royal entertainment they have given us today.

Seconded by Dr. Loveland, Clifton Springs. Carried.

THE PRESIDENT—Our meeting today has been an auspicious and happy one, successful both from a scientific and social standpoint. I wish to thank the members of the committee on arrangements and Dr. Gerin especially for the kind manner in which they have entertained the members of the association ; I also wish to thank the members of the various committees who have entered actively into the spirit of the work ; and I also wish to thank the members of the association for the kind attention which they have given their presiding officer.

If there is no further business, we stand adjourned, to meet on the third Tuesday in October, 1899, at Syracuse, N. Y.

